# Estimating the change in pleural pressure using the change in central venous pressure in various clinical scenarios: a pig model study

**DOI:** 10.1186/s40635-023-00590-8

**Published:** 2024-01-15

**Authors:** Miyako Kyogoku, Soichi Mizuguchi, Taku Miyasho, Yusuke Endo, Yu Inata, Kazuya Tachibana, Yuji Fujino, Kazuto Yamashita, Muneyuki Takeuchi

**Affiliations:** 1 Department of Intensive Care, Osaka Women’s and Children’s Hospital, 840 Murodo-Cho, Izumi, Osaka 594-1101 Japan; 2 Department of Emergency and Critical Care Center, Kyushu University, Fukuoka, Japan; 3 Laboratory of Animal Biological Responses, Department of Veterinary Science School of Veterinary Medicine, Rakuno Gakuen University, Hokkaido, Japan; 4 Laboratory for Critical Care, Department of Emergency Medicine-Cardio Pulmonary, Feinstein Institutes for Medical Research, Manhasset, NY USA; 5 Department of Anesthesiology, Osaka Women’s and Children’s Hospital, Osaka, Japan; 6 Department of Anesthesiology and Intensive Care Medicine, Osaka University Graduate School of Medicine, Osaka, Japan; 7 Department of Anesthesiology, Rakuno Gakuen University, Hokkaido, Japan

**Keywords:** Abdominal pressure, Acute respiratory distress syndrome, Animal model, Central venous pressure, Esophageal pressure, Intravascular volume, Mechanical ventilation, Pleural pressure, Respiratory failure, Transpulmonary pressure

## Abstract

**Background:**

We have previously reported a simple correction method for estimating pleural pressure (Ppl) using central venous pressure (CVP). However, it remains unclear whether this method is applicable to patients with varying levels of intravascular volumes and/or chest wall compliance. This study aimed to investigate the accuracy of our method under different conditions of intravascular volume and chest wall compliance.

**Results:**

Ten anesthetized and paralyzed pigs (43.2 ± 1.8 kg) were mechanically ventilated and subjected to lung injury by saline lung lavage. Each pig was subjected to three different intravascular volumes and two different intraabdominal pressures. For each condition, the changes in the esophageal pressure (ΔPes) and the estimated ΔPpl using ΔCVP (cΔCVP-derived ΔPpl) were compared to the directly measured change in pleural pressure (Δd-Ppl), which was the gold standard estimate in this study. The cΔCVP-derived ΔPpl was calculated as κ × ΔCVP, where “κ” was the ratio of the change in airway pressure to the change in CVP during the occlusion test. The means and standard deviations of the Δd-Ppl, ΔPes, and cΔCVP-derived ΔPpl for all pigs under all conditions were 7.6 ± 4.5, 7.2 ± 3.6, and 8.0 ± 4.8 cmH_2_O, respectively. The repeated measures correlations showed that both the ΔPes and cΔCVP-derived ΔPpl showed a strong correlation with the Δd-Ppl (ΔPes: r = 0.95, p < 0.0001; cΔCVP-derived ΔPpl: r = 0.97, p < 0.0001, respectively). In the Bland–Altman analysis to test the performance of the cΔCVP-derived ΔPpl to predict the Δd-Ppl, the ΔPes and cΔCVP-derived ΔPpl showed almost the same bias and precision (ΔPes: 0.5 and 1.7 cmH_2_O; cΔCVP-derived ΔPpl: − 0.3 and 1.9 cmH_2_O, respectively). No significant difference was found in the bias and precision depending on the intravascular volume and intraabdominal pressure in both comparisons between the ΔPes and Δd-Ppl, and cΔCVP-derived ΔPpl and Δd-Ppl.

**Conclusions:**

The CVP method can estimate the ΔPpl with reasonable accuracy, similar to Pes measurement. The accuracy was not affected by the intravascular volume or chest wall compliance.

**Supplementary Information:**

The online version contains supplementary material available at 10.1186/s40635-023-00590-8.

## Background

Limiting transpulmonary pressure (P_L_) has been proposed as an integral component of lung protective strategies for the management of acute respiratory distress syndrome (ARDS) [1]. Deriving P_L_ requires the measurement of esophageal pressure (Pes) as a surrogate for pleural pressure (Ppl) by using an esophageal balloon catheter [2]. However, the measurement of Pes is complicated because of several technical issues, including the correct positioning of the esophageal catheter and the feasibility of obtaining accurate measurements [3]. This explains why the measurement of Pes is not widely implemented in clinical settings [4, 5]. Therefore, it is important to establish a new and easier method for estimating Ppl without using an esophageal balloon.

The validity of using the change in the central venous pressure (ΔCVP) as a surrogate of the change in Ppl (ΔPpl) has been examined for decades [6, 7]. However, several studies have reported that the ΔCVP does not always accurately reflect the ΔPpl [8]. To address this limitation, we have developed a simple method of correcting ΔCVP [9, 10]. Briefly, we employed the change in airway pressure during an occlusion test (OT) to “calibrate” ΔCVP, aiming to mitigate its inherent inaccuracies. During an OT, where the lung gas volume remains the same, the change in airway pressure (ΔPaw) serves as a reliable indicator of ΔPpl [3]. In a previous study, we confirmed that the plateau P_L_ obtained using our correction method was close to the P_L_ values, which was measured by esophageal balloon in small children [9]. However, it remains unknown whether our method can be directly applied to patients, including adults, with various lung mechanics, including the chest wall to lung compliance ratio. The state of intravascular volume is also well known to affect the correlation between the ΔCVP and the change in esophageal pressure (ΔPes) [11], but this has not been investigated.

We hypothesized that our correction method using the ΔCVP would be applicable to passively ventilated patients with various chest wall compliances (Ccw) and volume statuses. To test our hypothesis, we compared the Ppl derived from our correction method with the directly measured intrapleural pressure (d-Ppl) in pigs with various Ccw and volume statuses.

## Methods

This study was approved by the Animal Care and Use Committee of Rakuno Gakuen University (no. VH17B6). The animal care and handling were performed in accordance with the guidelines of the National Institutes of Health, and adequate measures were taken to minimize the animals’ pain and discomfort.

### Animal preparation

Ten female pigs (LWD; ternary species) with an age of 3 months and weight of 43.2 ± 1.8 kg were used in the study. Food and water were withheld from the pigs for 12 h before experiment initiation. Then, 10 pigs were pre-medicated with intramuscular injection of medetomidine hydrochloride (40.0 μg/kg), midazolam (0.2 mg/kg), and butorphanol tartrate (0.2 mg/kg), and tracheal intubated after induction of anesthesia using propofol. General anesthesia was maintained with propofol (3.0–5.0 mg/kg/h). Following the induction of anesthesia, the pigs were placed in a dorsal recumbent position. Lactated Ringer’s solution (LRS; Solulact®, Terumo Co., Tokyo, Japan) was infused at 10 mL/kg/h through a 22-gauge catheter placed in the right marginal ear vein. A dedicated veterinarian assessed the depth of anesthesia regularly by checking the motor or hemodynamic response to a painful stimulus.

All pigs were mechanically ventilated (Servo-air, Getinge Group, Istanbul, Türkiye) in a volume-control mode after intravenous administration of 0.1 mg/kg vecuronium, followed by a constant-rate infusion at 0.6 mg/kg/h administered via the 22-gauge catheter in the left marginal ear vein. The ventilation settings were 8–10 mL/kg tidal volume with a 6 cmH_2_O positive end expiratory pressure (PEEP). The fraction of inspired oxygen (F_I_O_2_) was set at 0.4, with an inspiration: expiration ratio of 1:1.5–1:2.0, and the respiratory rate was adjusted to maintain the end tidal carbon dioxide (EtCO_2_) at 40–50 mmHg. Body temperature was maintained at 37–37.5 °C using a heating pad.

### Induction of lung injury

Lung injury was induced with bilateral lung lavage with 500 mL of isotonic saline warmed to 39 °C twice at a 15-min interval. The settings of mechanical ventilation between lavages were PEEP of 0 cmH_2_O, tidal volume of 500 mL, at a respiratory rate of 10 times/min, and F_I_O_2_ of 1.0, under the volume control ventilation. Following the lung lavages, a 60-min stabilization period was used to adjust the ventilator settings.

### Instrumentation

Several intravascular catheters were inserted invasively after anesthesia induction with the pigs in a dorsal recumbent position. Hair was removed from the skin over the right jugular vein, right femoral artery, and right chest wall. Each site was prepared aseptically and desensitized by subcutaneous injection of approximately 1.0–2.0 mL of 2% lidocaine (Xylocaine; AstraZeneca KK, Japan). The right femoral artery was catheterized using a thermistor-tipped 4 Fr, 16 cm PiCCO catheter connected to a PiCCO2 monitor (Getinge, Sweden). A 12-gauge triple-lumen central venous catheter (CVC) (SMAC™ Plus; Medtronic, Dublin, Ireland) was inserted into the right jugular vein and positioned at the cranial end of the superior vena cava.

For the direct measurement of intrathoracic pressure, a catheter was inserted 12 cm (5 cm from the side hole) horizontally into the thoracic cavity from the right midaxillary line between the sixth and seventh ribs using a 12 Fr sump tube (SalemSump™ tube, Medtronic, Japan). A 16 Fr esophageal balloon catheter (AVEA Ventilator Esophageal Pressure Monitoring Tube; IMI, Saitama, Japan) was inserted through the mouth, and the balloon was inflated with 2 mL of air. The amount of air in the esophageal balloon was adjusted accordingly depending on the agreement with the ΔPaw during the subsequent OT. A pneumotachometer for measuring the airway pressure (Paw) and airway flow was inserted at the junction of the respirator circuit and the endotracheal tube. Finally, an 8 Fr urinary catheter was inserted to measure the bladder pressure as a surrogate for intra-abdominal pressure.

### Monitoring and recording

Additional file 1: Figure S1 shows the layout of the monitor. Esophageal and airway pressures were measured using an RSS100 (Hans Rudolph Inc., Shawnee, KS, USA) and recorded using LabChart (ADInstruments, Sydney, Australia) via Power Lab (ADInstruments, Australia). The central venous pressure (CVP) and d-Ppl were measured and recorded using LabChart via Power Lab. Bladder pressure was measured using a vital signs monitor (Fukuda Electronics, Tokyo, Japan) through pressure lines and transducers (Merit Medical Inc., South Jordan, UT, USA) filled with saline, and the mean pressure was recorded manually.

The pressures were calibrated using several pressure levels obtained from gas pressure generators or water columns. The zero level of the water-filled transducer was set at the midaxillary line. The pressure lines filled with saline were flushed before each measurement to prevent errors caused by air contamination. To monitor bladder pressure, we infused 20 mL of saline into the bladder before pressure measurement.

### Experimental protocol

**Fig. 1:**
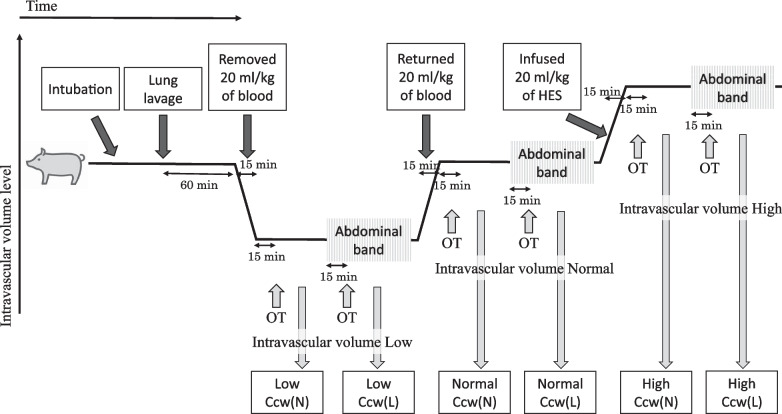
Schematic diagram of the protocol for the experiment. In each condition, waveform data were recorded during airway obstruction (OT) and mechanical ventilation to obtain the required data. *Ccw*, chest wall compliance; *OT*, occlusion test

The study design is summarized in Fig. 1.

## Manipulation of volume status and Ccw

The measurements were performed under six conditions (three levels of intravascular volumes and two levels of Ccw) for each pig. First, we removed 20 mL/kg of blood to simulate a “low” intravascular volume condition. The drawn blood was kept in a bag with citrate–phosphate-dextrose-adenine (TERUMO® Single Blood Bag, Terumo, Japan) at room temperature. Then, it was returned to simulate a “normal” intravascular volume condition. Finally, 20 mL/kg of hydroxyethyl starch was infused to simulate a “high” intravascular volume condition. Blood was removed and returned via the CVC over 15 min while carefully monitoring hemodynamics.

For each blood volume condition, the pigs were exposed to two Ccw conditions: normal Ccw (Ccw [N]) and low Ccw (Ccw [L]). The Ccw (L) condition was created by wrapping a band around the abdomen to increase the abdominal pressure to 15–20 mmHg. The Ccw (N) was defined as the Ccw condition without a band. Following every change in the intravascular volume or Ccw, the ventilator settings were adjusted to target a tidal volume of 8–10 mL/kg, EtCO_2_ of 40–50 mmHg, and oxygen saturation of 97–100%, and maintained for 15 min for stabilization.

After the stabilization, the arterial blood was sampled and analyzed. In addition, we measured the cardiac output, pulse pressure variation, stroke volume variation, and global end-diastolic volume (GEDV) to assess the intravascular volume using a PiCCO catheter. The bladder pressure was also recorded.

## Measurement of the ΔPes, ΔCVP, and the change in directly measured pleural pressure (Δd-Ppl)

**Fig. 2:**
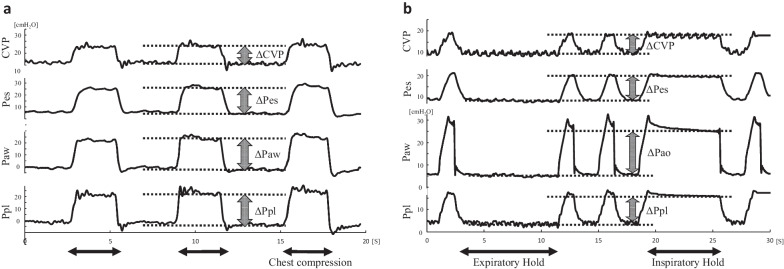
Pressure waveforms of CVP, Pes, Paw, and Ppl. Pressure waveforms during the occlusion test (**a**) and mechanical ventilation (**b**). **a** During chest compressions in the occlusion test, the waveforms of Pes and Paw fluctuated similarly; the ratio of the ΔPaw to the ΔCVP was determined and expressed as “κ”. **b** During mechanical ventilation, the ΔPpl can be calculated by multiplying “κ” with the ΔCVP, assuming that the ratio of the ΔPpl to the ΔCVP during the occlusion test and mechanical ventilation is the same. *CVP*, central venous pressure; *Paw*, airway pressure; *Pes*, esophageal pressure; *Ppl*, pleural pressure; *ΔCVP*, change in central venous pressure; *ΔPaw*, change in airway pressure; *ΔPes*, change in esophageal pressure; *ΔPpl*, change in pleural pressure

All measurements were performed in the supine position. First, the ΔPaw, ΔPes, ΔCVP, and Δd-Ppl were measured during an OT (Fig. 2a). Specifically, we opened the airway to the atmosphere at the end of expiration, then closed it, and the rib cage was gently squeezed to increase the Paw by approximately 10 cmH_2_O. Next, while on the same ventilator settings prior to OT, the animals were stabilized for 5 min. The plateau pressure, peak inspiratory pressure, total PEEP, and tidal volume were recorded. Then, the ΔPes, ΔCVP, and Δd-Ppl were measured using inspiratory and expiratory holds (Fig. 2b). The obtained result of the Δd-Ppl or ΔPes was deemed correct when the ratio of the Δd-Ppl or ΔPes to ΔPaw was between 0.8 and 1.2 [12, 13]. If the ratio was outside this range, the following measurements were excluded from the primary analysis.

## Estimation of the ΔPpl using the ΔCVP and a correction method (cΔCVP-derived ΔPpl)

Using the previously reported correction method [9], the ΔPpl was estimated from the ΔCVP using our previously published method (cΔCVP-derived ΔPpl). Briefly, the ratio of the ΔPaw to the ΔCVP was obtained by OT in each condition (Fig. 2a). This ratio is expressed as “κ” and was presumably similar to the ratio of the ΔPpl to the ΔCVP because the ΔPaw should be equal to the ΔPpl during airway occlusion. Next, the ΔCVP was measured during mechanical ventilation. Assuming that the ratio of the ΔPpl to the ΔCVP during the OT and mechanical ventilation is similar, the cΔCVP-derived ΔPpl can be expressed as follows:

$$c\Delta CVP-derived\;\Delta Ppl=\kappa\times\Delta CVP\;during\;the\;mechanical\;ventilation,$$

where κ is the ratio of the ΔPaw to the ΔCVP during OT.

### Analysis and statistics

#### I. Overall comparison of the ΔPes, cΔCVP-derived ΔPpl, and Δd-Ppl

The primary analysis sought to compare the two ΔPpl estimation methods (ΔPes and cΔCVP-derived ΔPpl) with the gold standard estimate of the ΔPpl (Δd-Ppl) using scatter plots for the repeated measures correlations and Bland–Altman (BA) analysis.

#### II. Performance of the ΔPes and cΔCVP-derived ΔPpl to predict the Δd-Ppl in each intravascular volume condition

To assess the effects of changes in intravascular volume on the performance of the ΔPes and cΔCVP-derived ΔPpl to predict the Δd-Ppl, BA analysis was performed in the low, normal, and high intravascular volume conditions.

#### III. Performance of the ΔPes and cΔCVP-derived ΔPpl to predict the Δd-Ppl in each Ccw condition

To evaluate the effects of the changes in chest wall compliance, a BA analysis was performed in the Ccw (N) and Ccw (L) groups.

Data are expressed as means ± standard deviations or medians (interquartile ranges [IQR]), depending on data distribution. Statistical significance was set at p < 0.05. Analyses were performed using the R Programming software (R Core Team, Vienna, Austria; URL: https://www.R-project.org).

## Results

A total of 60 data sets were obtained from 10 pigs. Because some of the data sets were excluded due to OT failure or measurement failure, the ΔPes was matched with the Δd-Ppl for comparison in 42 conditions, the cΔCVP-derived ΔPpl with the Δd-Ppl in 53conditions, and the ΔPes with CVP in 40 conditions (Additional file 2: Figure S2). The clinical data for each condition are shown in Table 1. The means and standard deviations of the ΔPes, cΔCVP-derived ΔPpl, and Δd-Ppl were 7.2 ± 3.6 cmH_2_O, 8.0 ± 4.8 cmH_2_O, and 7.6 ± 4.5 cmH_2_O, respectively (Table 2).

**Table 1:** Variables measured for each condition

	Low	Low	Normal	Normal	High	High	all
	Ccw(N)	Ccw(L)	Ccw(N)	Ccw(L)	Ccw(N)	Ccw(L)	
HR (bpm)	88.5 ± 22.3	84.7 ± 17.5	80.0 ± 13.1	81.9 ± 13.3	93.5 ± 13.4	99.0 ± 14.7	87.9 ± 16.8
mBP (mmHg)	58.8 ± 6.8	72.8 ± 5.1	102.9 ± 14.5	109.5 ± 10.4	117.0 ± 12.4	122.7 ± 13.3	97.3 ± 25.9
CVP (mmHg)	7.5 ± 1.0	11.7 ± 1.5	12.2 ± 1.2	15.7 ± 2.6	14.9 ± 2.6	19.7 ± 3.3	13.6 ± 4.4
abdominal pressure	7.5 ± 4.0	20.3 ± 3.2	7.3 ± 3.3	19.6 ± 3.3	8.1 ± 3.1	20.2 ± 3.2	13.8 ± 7.0
(mmHg)							
GEDV (ml)	496.8 ± 91.4	523.6 ± 104.4	689.7 ± 139.4	712.8 ± 79.7	746.3 ± 104.0	728.1 ± 116.1	649.6 ± 144.2
SVV (%)	18.4 ± 8.9	18.7 ± 6.2	11.9 ± 3.3	11.9 ± 3.3	7.0 ± 1.9	10.8 ± 5.8	13.1 ± 6.7
PPV (%)	24.4 ± 5.6	22.9 ± 7.0	10.1 ± 1.8	12.2 ± 3.3	8.1 ± 2.5	10.7 ± 3.7	14.7 ± 7.7
plat P (cmH_2_O)	19.1 ± 2.3	27.9 ± 5.4	20.7 ± 5.1	31.0 ± 6.9	23.3 ± 6.4	32.5 ± 6.3	25.8 ± 7.4
PEEP (cmH_2_O)	6.0 ± 0.0	6.0 ± 0.0	6.0 ± 0.0	6.0 ± 0.0	6.0 ± 0.0	6.0 ± 0.0	6.0 ± 0.0
F_I_O_2_	0.38 ± 0.04	0.39 ± 0.0	0.44 ± 0.20	0.40 ± 0.08	0.45 ± 0.18	0.41 ± 0.07	0.41 ± 0.12
TV (ml)	343.0 ± 30.6	342.6 ± 25.1	356.4 ± 44.7	341.9 ± 30.6	350.2 ± 28.9	345.4 ± 23.6	346.6 ± 30.4
PaO_2_ (mmHg)	145.2 ± 38.2	137.3 ± 44.7	188.9 ± 112.6	114.6 ± 47.5	145.6 ± 86.3	79.6 ± 28.6	135.2 ± 72.0
PaCO_2_ (mmHg)	45.2 ± 4.9	45.0 ± 4.6	47.7 ± 3.7	46.1 ± 6.0	47.4 ± 6.5	50.0 ± 7.6	46.9 ± 5.7
P/F	380.0 ± 82.5	351.6 ± 106.9	416.5 ± 81.1	295.6 ± 129.0	321.3 ± 122.2	201.1 ± 87.5	327.7 ± 120.7

The values are presented as means ± standard deviations

*CVP* central venous pressure, *GEDV* global end-diastolic volume, *HR* heart rate, *mBP* mean blood pressure, *PEEP* Positive end-expiratory pressure, *plat P* plateau pressure, *PPV* pulse pressure variation, *SVV* stroke volume variation, *TV* tidal volume

**Table 2:** Variables measured to obtain the change in pleural pressure

	Low	Low	Normal	Normal	High	High	all
	Ccw(N)	Ccw(L)	Ccw(N)	Ccw(L)	Ccw(N)	Ccw(L)	
Crs (ml/cmH_2_O)	26.9 ± 4.9	16.4 ± 3.8	26.0 ± 6.6	14.6 ± 3.8	22.2 ± 6.5	13.7 ± 3.3	20.0 ± 7.2
Ccw (ml/cmH_2_O)	100.6 ± 30.3	37.1 ± 18.3	81.7 ± 21.0	35.3 ± 14.7	82.8 ± 20.9	38.3 ± 19.1	62.5 ± 33.9
CL (ml/cmH_2_O)	36.4 ± 7.4	30.5 ± 11.0	34.9 ± 13.2	25.0 ± 13.7	27.3 ± 9.3	21.6 ± 8.8	34.4 ± 11.3
ΔPes/ΔPaw	0.9 ± 0.1	0.9 ± 0.1	0.9 ± 0.1	0.9 ± 0.1	0.9 ± 0.1	0.9 ± 0.0	0.9 ± 0.1
during OT							
ΔPaw/ΔCVP	1.7 ± 0.8	2.0 ± 1.3	2.6 ± 1.6	2.1 ± 1.2	1.9 ± 1.1	2.8 ± 1.6	2.2 ± 1.3
during OT (κ)							
Δd-Ppl (cmH_2_O)	3.7 ± 1.1	10.7 ± 3.8	4.5 ± 1.1	11.3 ± 4.8	4.5 ± 1.2	10.5 ± 3.6	7.6 ± 4.5
(n)	10	9	9	10	8	9	55
ΔPes (cmH_2_O)	4.0 ± 1.6	10.0 ± 2.7	4.7 ± 1.3	10.3 ± 3.6	4.1 ± 0.8	9.5 ± 1.6	7.2 ± 3.6
(n)	6	7	7	8	7	7	42
cΔCVP-derived ΔPpl (cmH_2_O)	3.8 ± 1.4	11.2 ± 3.4	4.7 ± 2.2	11.8 ± 5.6	4.2 ± 1.3	11.1 ± 2.8	8.0 ± 4.8
(n)	10	9	8	10	7	9	53

The values are presented as means ± standard deviations. Ccw and CL were calculated using d-Ppl

*Ccw* chest wall compliance; *CL* lung compliance; *Crs* respiratory system compliance; *d-Ppl* directly measured a pleural pressure; *OT* occlusion test; *ΔCVP* change in central venous pressure; *Δd-Ppl* change in directly measured pleural pressure; *ΔPaw* change in airway pressure; *ΔPes* change in esophageal pressure

### I. Overall comparison of the ΔPes, cΔCVP-derived ΔPpl, and Δd-Ppl

**Fig. 3:**
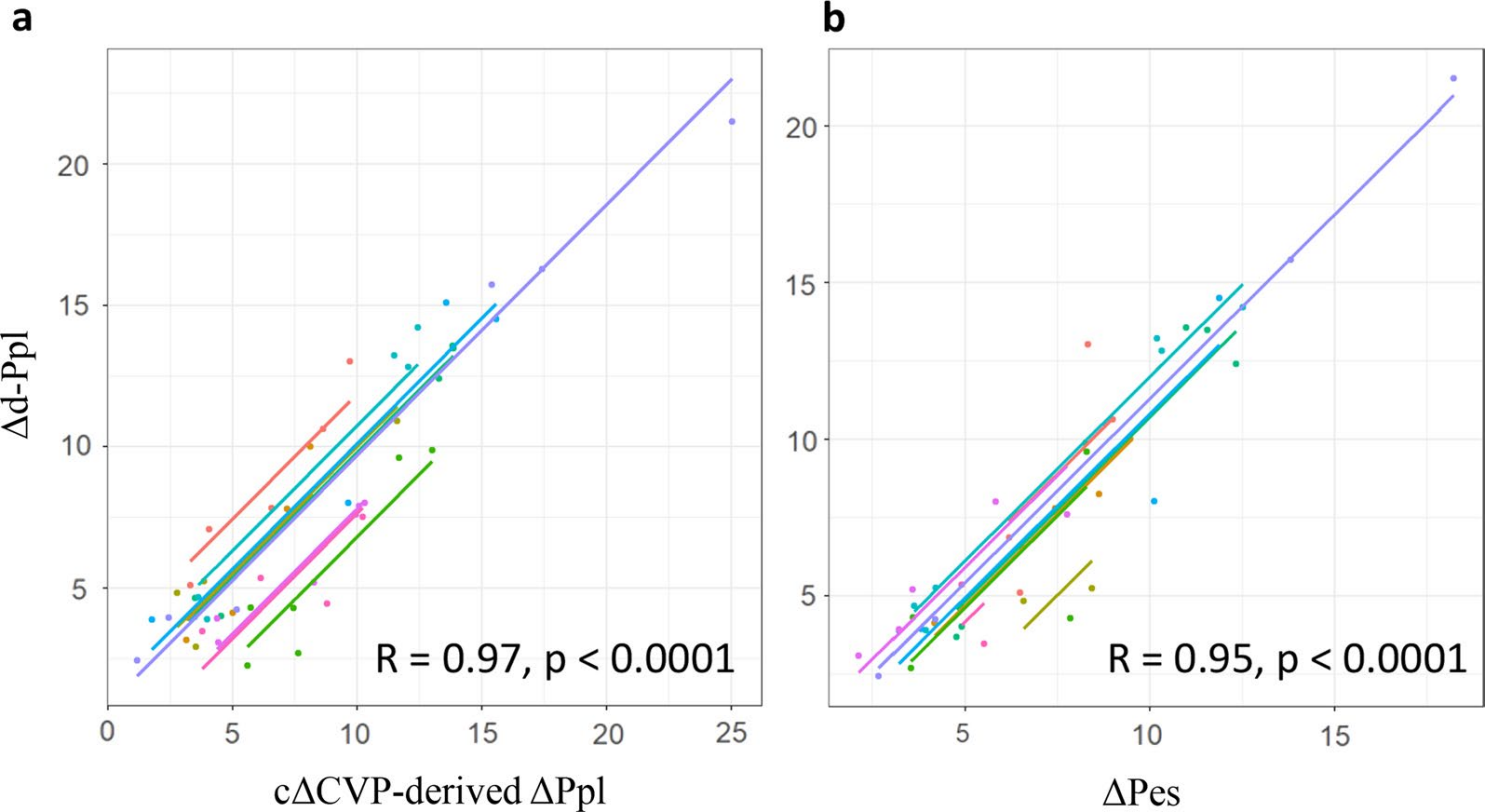
Comparison of two variables that reflect the ΔPpl. Scatter plots for the RMCORR between Δd-Ppl and the cΔCVP-derived ΔPpl (**a**), Δd-Ppl and the ΔPes (**b**), and the cΔCVP-derived ΔPpl and ΔPes (**c**). Correlation coefficients and adjusted P-values are shown for each comparison. For comparison, data from the same pig were colored differently, with a single color for all time points from the same pig. cΔCVP-derived *ΔPpl*, ΔPpl calculated using a corrected ΔCVP; *RMCORR*, repeated measures correlations; *ΔCVP*, change in central venous pressure; *Δd-Ppl*, change in directly measured pleural pressure; *ΔPes*, change in esophageal pressure

**Fig. 4:**
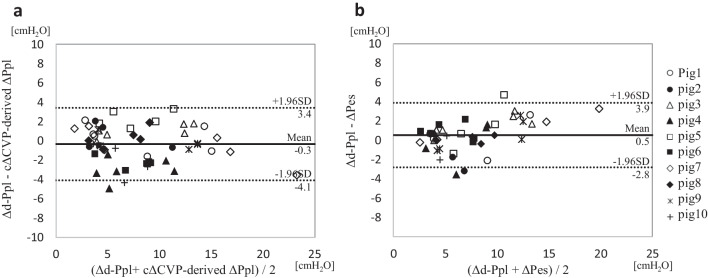
The Bland–Altman analysis of two variables reflecting the ΔPpl. The Bland–Altman analysis for the agreement between the cΔCVP-derived ΔPpl and Δd-Ppl (**a**) and between the ΔPes and Δd-Ppl (**b**). Solid lines indicate bias (a: − 0.3 cmH_2_O, b: 0.5 cmH_2_O). Broken lines indicate ± 1.96 standard deviation of the bias (a: 3.4 and − 4.1 cmH_2_O; and b: 3.9 and − 2.8 cmH_2_O). For comparison, data from the same pigs were marked with the same marker. cΔCVP-derived ΔPpl, ΔPpl calculated using a corrected ΔCVP; *Δd-Ppl*, change in directly measured pleural pressure; *ΔPes*, change in esophageal pressure; *ΔPpl*, change in pleural pressure

Figure 3 shows the correlation between repeated measures. Both the ΔPes and cΔCVP-derived ΔPpl showed a strong correlation with the Δd-Ppl (ΔPes: r = 0.95, p < 0.0001; cΔCVP-derived ΔPpl: r = 0.97, p < 0.0001, respectively). In the BA analysis to test the performance of the ΔPes and cΔCVP-derived ΔPpl to predict the Δd-Ppl, the ΔPes and cΔCVP-derived ΔPpl showed almost the same bias and precision (ΔPes: 0.5 and 1.7 cmH_2_O; cΔCVP-derived ΔPpl: − 0.3 and 1.9 cmH_2_O, respectively) (Fig. 4).

### II. Performance of the ΔPes and cΔCVP-derived ΔPpl to predict the Δd-Ppl in each intravascular volume condition

**Fig. 5:**
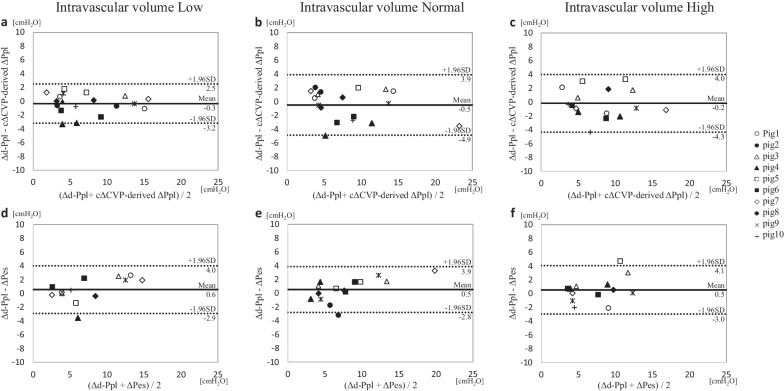
The Bland–Altman analysis of each variable is classified by intravascular volume. The a, b, and c figures show the Bland**–**Altman analysis for the agreement between the cΔCVP-derived ΔPpl and Δd-Ppl in the intravascular volume condition set to low (**a**), normal (**b**), and high (**c**), respectively. The d, e, and f figures show the Bland**–**Altman analysis for the agreement between the ΔPes and Δd-Ppl in the intravascular volume condition set to low (**d**), normal (**e**), and high (**f**), respectively. Solid lines indicate bias (a: − 0.3 cmH_2_O, b: − 0.5 cmH_2_O, c: − 0.2 cmH_2_O, d: 0.6 cmH_2_O, e: 0.5 cmH_2_O, and f: 0.5 cmH_2_O). Broken lines indicate ± 1.96 standard deviation of the bias (a: 2.5 and − 3.2 cmH_2_O; b: 3.9 and − 4.9 cmH_2_O; c: 4.0 and − 4.3 cmH_2_O; d: 4.0 and − 2.9 cmH_2_O; e: 3.9 and − 2.8 cmH_2_O; and f: 4.1 and − 3.0 cmH_2_O). For comparison, data from the same pigs were marked with the same marker. cΔCVP-derived ΔPpl, ΔPpl calculated using a corrected ΔCVP; *Δd-Ppl*, change in directly measured pleural pressure; *ΔPes*: change in esophageal pressure

As a result of changing the intravascular volume, parameters, such as the CVP and GEDV, in each group changed. Figure 5 shows the results of the BA analyses to test the performance of the ΔPes and cΔCVP-derived ΔPpl to predict the Δd-Ppl in low, normal, and high intravascular volume conditions. The biases and precisions were similar among the three different intravascular volume conditions.

### III. Performance of the ΔPes and cΔCVP-derived ΔPpl to predict the Δd-Ppl in each Ccw condition

**Fig. 6:**
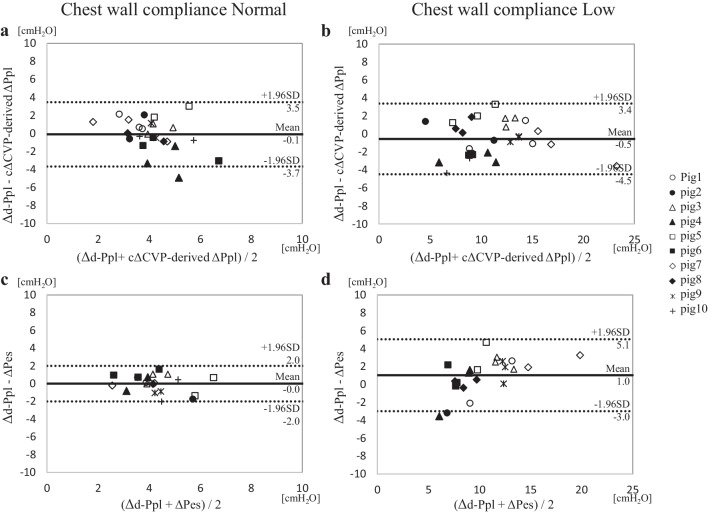
The Bland–Altman analysis of each variable classified by chest wall compliance. The a and b figures show the Bland**–**Altman analysis for the agreement between the cΔCVP-derived ΔPpl and Δd-Ppl in normal chest wall compliance (**a**) and low chest wall compliance (**b**). The c and d figures show the Bland**–**Altman analysis for the agreement between the ΔPes and Δd-Ppl in normal chest wall compliance (**c**) and low chest wall compliance (**d**). Solid lines indicate bias (a: − 0.1 cmH_2_O; b: − 0.5 cmH_2_O; c: 0.0 cmH_2_O; d: 1.0 cmH_2_O). Broken lines indicate ± 1.96 standard deviation of the bias (a: 3.5 and − 3.7 cmH_2_O; b: 3.4 and − 4.5 cmH_2_O; c: 2.0 and − 2.0 cmH_2_O; d: 5.1 and − 3.0 cmH_2_O). For comparison, data from the same pigs were marked with the same marker. cΔCVP-derived ΔPpl, ΔPpl calculated using a corrected ΔCVP; *Δd-Ppl*, change in directly measured pleural pressure; *ΔPes*, change in esophageal pressure

With the abdominal bandage, the Ccw changed from 89.0 ± 25.6 mL/cmH_2_O to 36.8 ± 16.8 mL/cmH_2_O. Figure 6 shows the results of the BA analyses to test the performance of the ΔPes and cΔCVP-derived ΔPpl to predict the Δd-Ppl in the Ccw (N) and Ccw (L) conditions. The biases and precisions were similar for the two different Ccw conditions.

## Discussion

In this study, we examined the accuracy of our method under different conditions of intravascular volume and chest wall compliance. Our group previously reported that the ΔPpl could be estimated from the ΔCVP using a correction method in pediatric patients [9, 10]. However, these studies have several limitations in terms of generalizability. It is also known that the relationship between the ΔPpl and ΔCVP (corresponding to “κ” in this study) is influenced by the mean CVP value altered by infusion, but our previous study did not examine this point [14]. Therefore, in the present study, the validity of our method was tested in adult-sized pigs with various Ccw and intravascular volumes. Our method of estimating ΔPpl using ΔCVP is not highly accurate. Nonetheless, it is as accurate as Pes, which is commonly considered the gold standard technique, regardless of the intravascular volume and Ccw. Furthermore, cΔCVP-derived ΔPpl estimated using our method could better represent Δd-Ppl than the original ΔCVP (Additional file 3: Figure S3).

Esophageal pressure balloon pleural pressure measurement presents several technical challenges for correct positioning and accurate measurement. For example, determining the appropriate amount of air injected into the balloon can be challenging. Thus, various studies have explored calibration methods for balloons and measurement techniques that do not rely on the use of a balloon [15, 16]. Additionally, many studies have examined whether the ΔCVP can be used to estimate the ΔPpl without an esophageal balloon catheter. Several studies have shown a correlation between the ΔCVP and ΔPes [6, 7, 17], while others have reported conflicting results [8, 11].

One reason for the contradictory results is that the ΔCVP values are influenced by intravascular volume as well as by the ΔPpl. Therefore, we hypothesized that intravascular volume could also influence the accuracy of the ΔPpl prediction equation. We anticipated that the effect of Ppl on CVP would be more pronounced when intravascular volume was lower, given that CVP fluctuation is indicative of fluid responsiveness [18]. Hence, in this study, we investigated whether the intravascular volume affected the accuracy of our correction method by changing it to three levels (low, normal, and high) by blood removal or transfusion (Table 1). Transfusion increased intravascular volume and CVP, as indicated by indices such as GEDV, whereas blood removal decreased them. Contrary to our prediction, our correction methods could accurately and precisely estimate ΔPpl in all groups with varying intravascular volumes. In our study, a correction factor “κ” obtained by OT was applied to subsequent calculations of cΔCVP-derived ΔPpl immediately after obtaining “κ”. However, further investigation is needed to determine the duration for which “κ” remains unchanged after OT is performed.

In this study, to create a model of lower-than-normal chest wall compliance, we wrapped an abdominal bandage around the patient to create a situation similar to abdominal compartment syndrome. The abdominal bandage led to an elevation in CVP (Table 1), potentially influencing the association between ΔPpl and ΔCVP. However, as elevated CVP does not affect the accuracy of our correction method, as discussed above, we believe that we have demonstrated that our correction method can be used even under conditions of decreased Ccw.

Our method of using CVP has several advantages over Ppl measurement using esophageal balloon catheters. First, this method uses a CVC that has already been inserted and is, therefore, less costly, while esophageal balloon catheters are expensive and require practically dedicated devices. In a study by Tag et al., the CVP was measured in 45.6% of patients with ARDS or at risk of ARDS within 24 h of intensive care unit admission [19]. Second, our method using CVP can be used to estimate Ppl in patients with relative contraindications of esophageal balloon catheter insertion, such as esophageal varices, hiatal hernia, and bleeding diathesis. Finally, our method can be used as an alternative to esophageal pressure when the patient does not pass the OT. In the current study, 13 sets of data (24%) were excluded because the changes in Pes and Paw during OT differed by more than 20%. The high number of cases of failed OT in the present study may be due to the different shapes of the chest wall between pigs and humans [20]; however, we have experienced many cases of failed OT in children as well [9, 10]. A post-hoc analysis that included cases that did not pass the OT demonstrated that the correlation between the cΔCVP-derived ΔPpl and Δd-Ppl remained significant in the present experiment (Additional file 4: Figure S4).

This study had several limitations. First, it was an animal study, and our setting may differ from that in actual clinical practice. However, the direct measurement of Ppl and a protocol for actively changing the intravascular volume are impossible in humans. Second, although we have already reported that our method can be used in spontaneously breathing pediatric patients [10], it remains unclear whether our method can be applied to spontaneous breathing in adults with ARDS, as this study was performed only in paralyzed pigs. This could be an area of potential interest for future research. Third, our method does not provide the absolute value of pleural pressure that clinicians may sometimes require. However, even with the gold standard esophageal pressure, caution is warranted when relying on absolute values [4]. On the contrary, utilizing the elastance method can estimate absolute value of the transpulmonary plateau pressure, making it useful in clinical practice [1, 9]. Finally, our method requires an airway opening procedure when performing OT. Furthermore, airway opening in patients with severe ARDS is a procedure that should be avoided if possible, as it leads to alveolar collapse. Airway opening also increases the risk of exposure to viral droplets and aerosols. It remains unknown if and how the airway closure caused by an airway opening procedure changes the relationship between airway pressure and pleural pressure during OT. If airway closure occurred with airway opening during this study, the variation in pleural pressure may not have been transmitted correctly to the airway pressure, thereby potentially influencing the results of this study. Although there are reports that OT can be performed under PEEP [12], it is unclear at this time whether accuracy can be maintained with κ obtained under PEEP and is a subject for future research.

## Conclusions

In conclusion, our method using the ΔCVP can estimate the ΔPpl with the same accuracy as ΔPes in mechanically ventilated pigs with muscle paralysis during acute respiratory failure. In addition, our method can estimate the ΔPpl regardless of the intravascular volume and Ccw.

### Supplementary Information

**Additional file 1: Figure S1.** Schematic of the experimental setup for monitoring. Pigs were immobilized under anesthesia, placed on a ventilator, and monitored for airway pressure, esophageal pressure, CVP, Ppl, and PiCCO. CVP, central venous pressure; Ppl, pleural pressure.

**Additional file 2: Figure S2.** Consolidated Standards of Reporting diagram showing eligible, included, and excluded data. In total, 60 data were obtained from 10 pigs. ^*1^Five data were excluded because the Paw or Ppl measurements failed. These data determined that data measurement was incorrect since the ratio of the ΔPpl to ΔPaw should be between 0.8 and 1.2. ^*2^14 esophageal pressure data were excluded because the ratio of the ΔPes to ΔPaw was not between 0.8 and 1.2. ^*3^Two CVP data points were excluded owing to CVP measurement failure. Finally, 55 data of chest pressure data points, 42 data of esophageal pressure data points, and 53 data of CVP data points were analyzed. CVP, central venous pressure; Paw, airway pressure; Pes, esophageal pressure; Ppl, pleural pressure; Δd-Ppl, change in directly measured pleural pressure; ΔPaw, change in airway pressure; ΔPes, change in esophageal pressure

**Additional file 3: Figure S3.** Comparison of the ΔCVP, cΔCVP-derived ΔPpl, and Δd-Ppl. Scatter plots between the ΔCVP, cΔCVP-derived ΔPpl, and Δd-Ppl. The broken line represents a simple linear regression line for ΔCVP, whereas the solid line represents a simple linear regression line for cΔCVP-derived. cΔCVP-derived ΔPpl, corrected change in central venous pressure; ΔCVP, change in central venous pressure; Δd-Ppl, change in directly measured pleural pressure

**Additional file 4: Figure S4.** Comparison of the cΔCVP-derived ΔPpl and ΔPes with all data. Scatter plots for the RMCORR between the cΔCVP-derived ΔPpl and ΔPes. Correlation coefficients and adjusted P-values are presented for each comparison. For comparison, data from the same pig were colored differently, with a single color for all time points from the same pig. These results include all data of the Pes that did not pass the OT. Nevertheless, the correlation between Δd-Ppl and the ΔPes remained significant. cΔCVP-derived ΔPpl, corrected change in central venous pressure; OT, occlusion test; Pes, esophageal pressure; RMCORR, repeated measures correlations; Δd-Ppl, change in directly measured pleural pressure; ΔPes, change in esophageal pressure

## Data Availability

The datasets used and/or analyzed during the current study are available from the corresponding author on reasonable request.

## Abbreviations

ARDS: Acute respiratory distress syndrome
BA: Bland–Altman
Ccw: Chest wall compliance
CVC: Central venous catheter
CVP: Central venous pressure
cΔCVP-derived ΔPpl: ΔPpl calculated using a corrected ΔCVP
d-Ppl: Directly measured pleural pressure
EtCO_2_: The end tidal carbon dioxide
F_I_O_2_: Fraction of inspired oxygen
GEDV: Global end-diastolic volume
OT: Occlusion test
Paw: Airway pressure
PEEP: Positive End Expiratory Pressure
Pes: Esophageal pressure
P_L_: Transpulmonary pressure
Ppl: Pleural pressure
ΔCVP: The change in central venous pressure
Δd-Ppl: The change in directly measured pleural pressure
ΔPaw: The change in airway pressure
ΔPes: The change in esophageal pressure
ΔPpl: The change in pleural pressure
