# Supplementary material for: Estimating the change in pleural pressure using the change in central venous pressure in various clinical scenarios: a pig model study
Source: Intensive Care Med Exp. 2024 Jan 15;12:4. doi: 10.1186/s40635-023-00590-8 (PMC10789683; doi:10.1186/s40635-023-00590-8)
Supplement: Supplementary file 1 — Additional file 1: Figure S1. Schematic of the experimental setup for monitoring. Pigs were immobilized under anesthesia, placed on a ventilator, and monitored for airway pressure, esophageal pressure, CVP, Ppl, and PiCCO. CVP, central venous pressure; Ppl, pleural pressure. [file 40635_2023_590_MOESM1_ESM.pptx]

## Slide 1
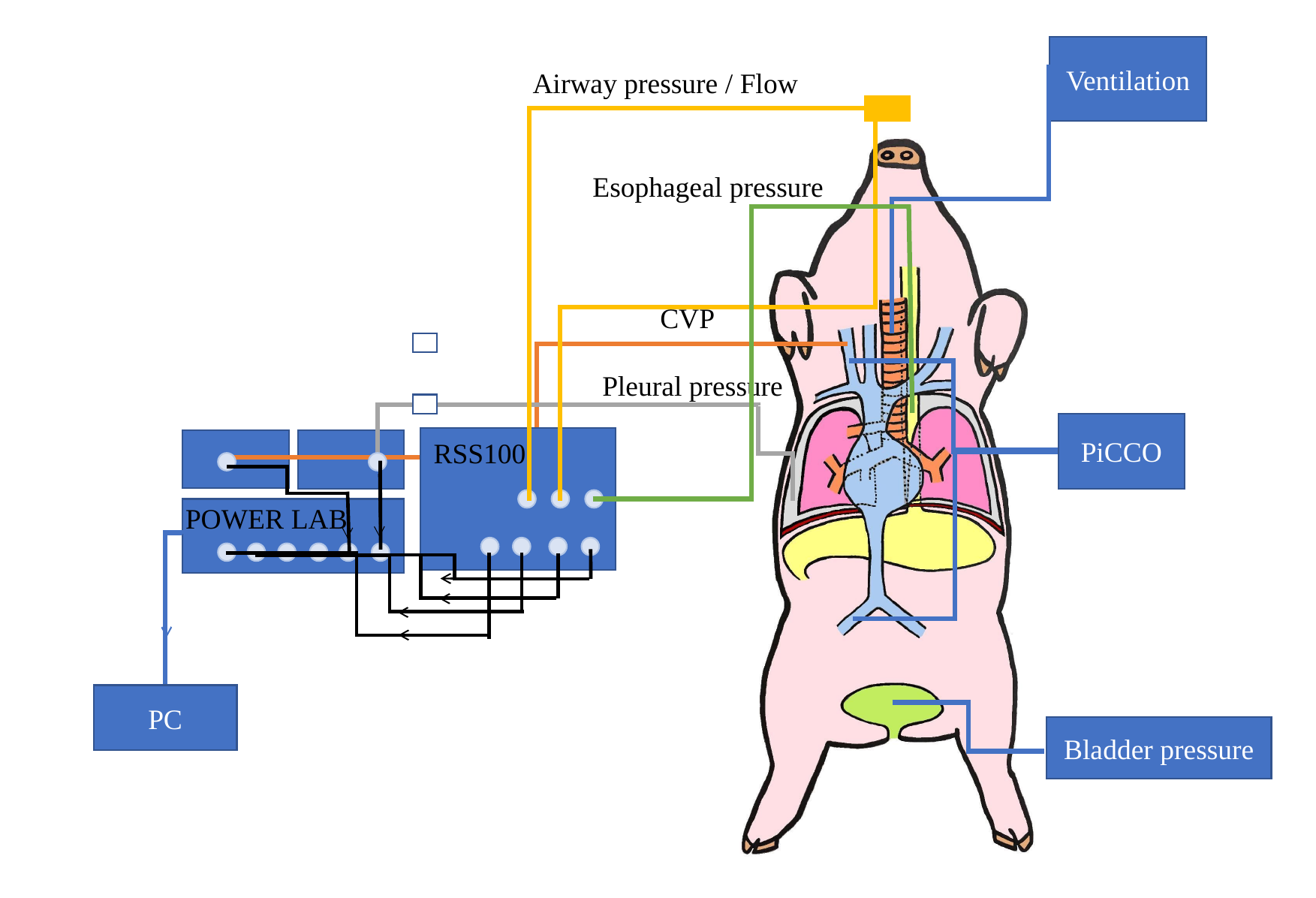

Ventilation
Airway pressure / Flow
Esophageal pressure
CVP
Pleural pressure
PiCCO
RSS100
POWER LAB
PC
Bladder pressure
