# Supplementary material for: Estimating the change in pleural pressure using the change in central venous pressure in various clinical scenarios: a pig model study
Source: Intensive Care Med Exp. 2024 Jan 15;12:4. doi: 10.1186/s40635-023-00590-8 (PMC10789683; doi:10.1186/s40635-023-00590-8)
Supplement: Supplementary file 2 — Additional file 2: Figure S2. Consolidated Standards of Reporting diagram showing eligible, included, and excluded data. In total, 60 data were obtained from 10 pigs. *1Five data were excluded because the Paw or Ppl measurements failed. These data determined that data measurement was incorrect since the ratio of the ΔPpl to ΔPaw should be between 0.8 and 1.2. *214 esophageal pressure data were excluded because the ratio of the ΔPes to ΔPaw was not between 0.8 and 1.2. *3Two CVP data points were excluded owing to CVP measurement failure. Finally, 55 data of chest pressure data points, 42 data of esophageal pressure data points, and 53 data of CVP data points were analyzed. CVP, central venous pressure; Paw, airway pressure; Pes, esophageal pressure; Ppl, pleural pressure; Δd-Ppl, change in directly measured pleural pressure; ΔPaw, change in airway pressure; ΔPes, change in esophageal pressure [file 40635_2023_590_MOESM2_ESM.pptx]

## Slide 1
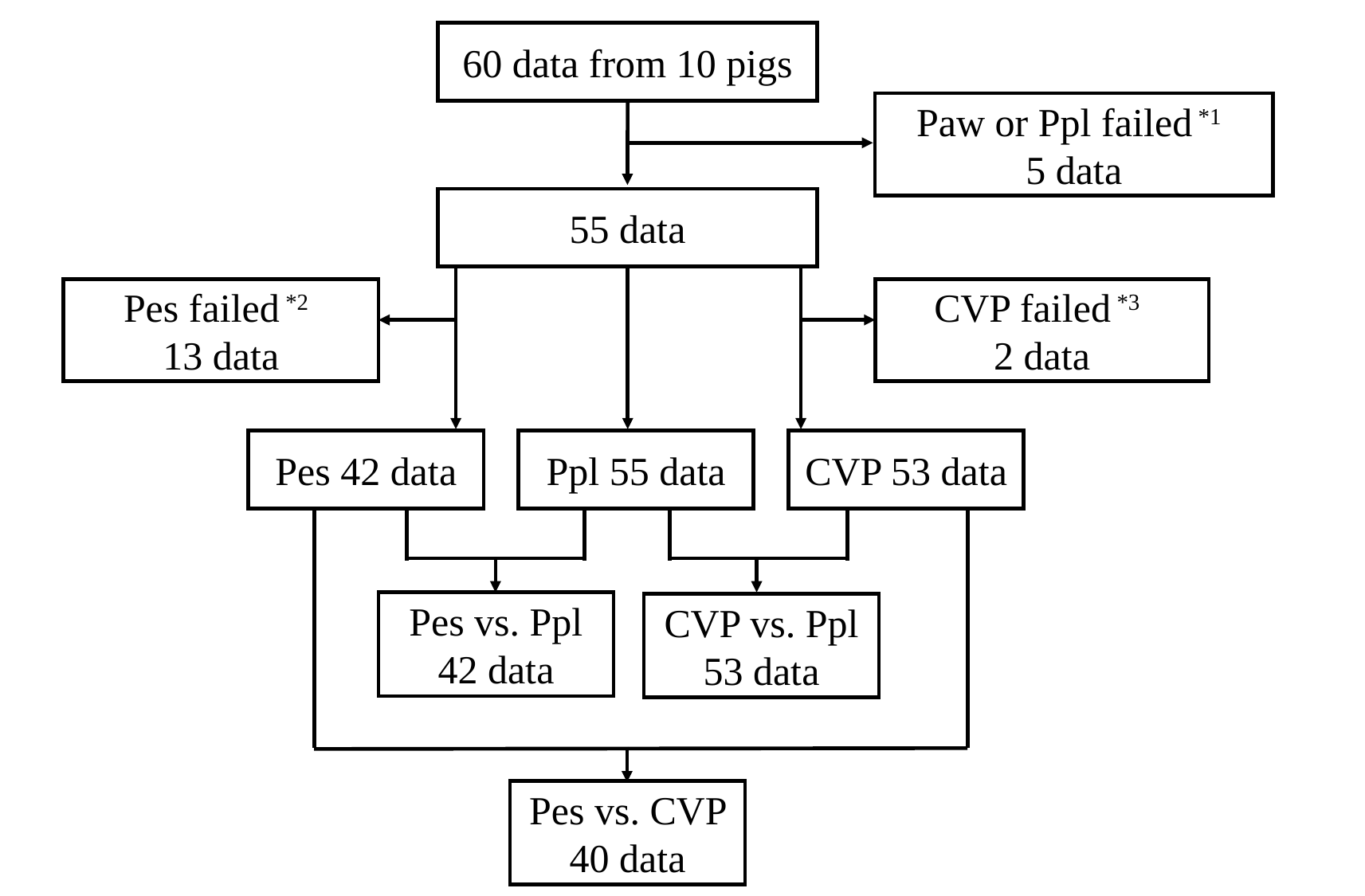

60 data from 10 pigs
Paw or Ppl failed *1
5 data
55 data
Pes failed *2
13 data
CVP failed *3
2 data
Pes 42 data
Ppl 55 data
CVP 53 data
Pes vs. Ppl 42 data
CVP vs. Ppl 53 data
Pes vs. CVP 40 data
