# Supplementary figures and images for: Estimating the change in pleural pressure using the change in central venous pressure in various clinical scenarios: a pig model study
Source: Intensive Care Med Exp. 2024 Jan 15;12:4. doi: 10.1186/s40635-023-00590-8 (PMC10789683; doi:10.1186/s40635-023-00590-8)

## Slide 1
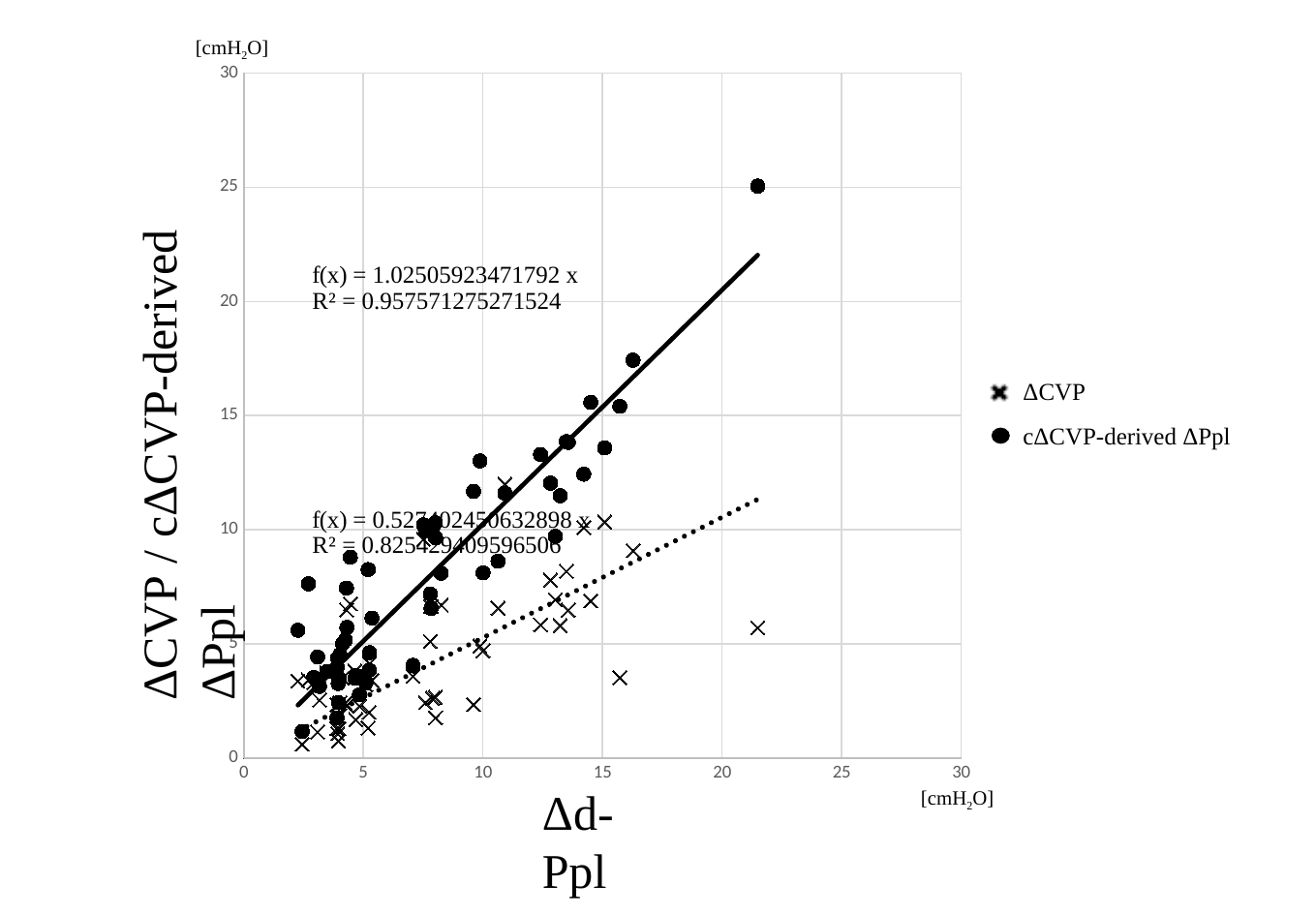

[cmH2O]
### Chart
| Category | CVP | 補正CVP |
|---|---|---|ΔCVP / cΔCVP-derived ΔPpl
ΔCVP
cΔCVP-derived ΔPpl
Δd-Ppl
[cmH2O]

Supplement: Supplementary file 3 — Additional file 3: Figure S3. Comparison of the ΔCVP, cΔCVP-derived ΔPpl, and Δd-Ppl. Scatter plots between the ΔCVP, cΔCVP-derived ΔPpl, and Δd-Ppl. The broken line represents a simple linear regression line for ΔCVP, whereas the solid line represents a simple linear regression line for cΔCVP-derived. cΔCVP-derived ΔPpl, corrected change in central venous pressure; ΔCVP, change in central venous pressure; Δd-Ppl, change in directly measured pleural pressure [file 40635_2023_590_MOESM3_ESM.pptx]

## Slide 1
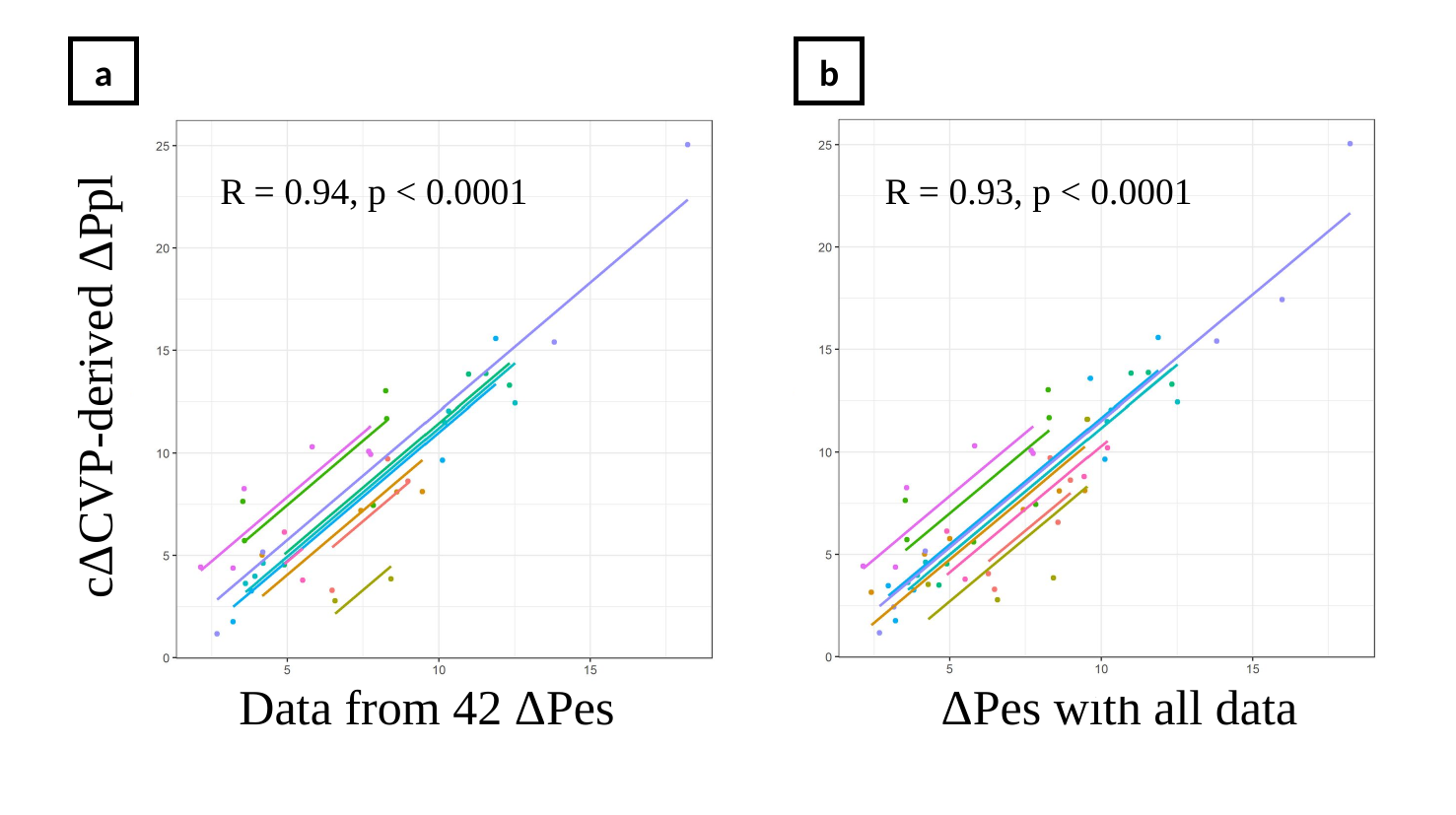

cΔCVP-derived ΔPpl
a
b
R = 0.94, p < 0.0001
R = 0.93, p < 0.0001
ΔPes with all data
Data from 42 ΔPes

Supplement: Supplementary file 4 — Additional file 4: Figure S4. Comparison of the cΔCVP-derived ΔPpl and ΔPes with all data. Scatter plots for the RMCORR between the cΔCVP-derived ΔPpl and ΔPes. Correlation coefficients and adjusted P-values are presented for each comparison. For comparison, data from the same pig were colored differently, with a single color for all time points from the same pig. These results include all data of the Pes that did not pass the OT. Nevertheless, the correlation between Δd-Ppl and the ΔPes remained significant. cΔCVP-derived ΔPpl, corrected change in central venous pressure; OT, occlusion test; Pes, esophageal pressure; RMCORR, repeated measures correlations; Δd-Ppl, change in directly measured pleural pressure; ΔPes, change in esophageal pressure [file 40635_2023_590_MOESM4_ESM.pptx]
